# Supplementary material for: Are carbon emissions trading and green financial instruments synergistic? -Comprehensive quantitative research based on content analysis
Source: PLoS One. 2024 Mar 7;19(3):e0298601. doi: 10.1371/journal.pone.0298601 (PMC10919601; doi:10.1371/journal.pone.0298601)
Supplement: S1 Data — (ZIP) [file pone.0298601.s001.zip › Data/地级市Z_F工作报告.zip!/║■▒▒/╬Σ║║╩╨/└┤╫╘╣1⁄2╓┌║┼í╛┬φ┐╦╩2╛▌═°í┐_2014.docx]

各位代表：

　　现在，我代表市人民政府向大会报告工作，请予审议，并请市政协委员和其他列席人员提出意见。

　　一、2013年工作回顾

　　2013年，是实施“十二五”规划承上启下的关键年。面对国际环境错综复杂、国内经济下行压力较大的严峻形势，我们在省委、省政府和市委的坚强领导下，紧紧依靠全市人民，坚持以党的十八大、十八届三中全会和习近平总书记视察湖北武汉重要讲话精神为指引，坚持竞进提质、效速兼取不动摇，保持定力、从容应对，攻坚克难、砥砺前行，全面实施“五大计划”，大力推进美丽江城、幸福武汉建设，在建设国家中心城市和国际化大都市征程中迈出了新的步伐。

　　——综合实力再上新台阶。预计全市地区生产总值突破9000亿元，全口径财政收入超过2400亿元，全社会固定资产投资突破6000亿元，社会消费品零售总额超过3800亿元。

　　——工业倍增成效明显。规模以上工业总产值突破万亿元，单月产值突破千亿元。汽车产业产值突破2000亿元。产值过500亿元企业增加到4户，过百亿元企业达到15户，新增3户;80万吨乙烯、神龙三厂、联想武汉产业基地、武钢四冷轧等一批投资50亿元、100亿元以上重大项目建成投产，东风雷诺项目落户武汉，发展后劲显著增强。

　　——创新驱动发展步伐加快。实施重大科技成果转化专项86项。新增高新技术企业89家，累计900家，产值过百亿元高新技术企业达9家。高新技术产业产值5500亿元，占全市规模以上工业总产值的55%，比上年提高5个百分点。在全国高新区中，东湖高新区综合排名上升到第3位，知识创造和技术创新能力上升到第2位。

　　——交通枢纽功能不断提升。轨道交通4号线一期建成试运营，与轨道交通1号线、2号线一期构成“工”字形轨道交通架构，轨道交通连通三大火车站，可达五大长途客运站，高铁、城铁、地铁、长途客运实现快速换乘。以武汉为中心的“米”字形高铁网基本形成，构成武汉到郑州、长沙、南昌、合肥等中部省会城市2小时交通圈;到北京、上海、广州、西安等城市4-5小时交通圈;到重庆、宁波、青岛、厦门等城市8小时左右交通圈。武汉火车站成为目前全国高铁线路辐射最广、直达城市最多的高铁站，年旅客发送量突破千万人次。内陆港口首条近洋班轮——“泸汉台集装箱快班”开通。天河机场旅客吞吐量达到1570万人次。

　　——对外开放实现新突破。新引进世界500强企业23家，累计200家。新增国际和地区航线5条，累计25条;国际和地区旅客吞吐量突破100万人次，增长59%;国际和地区航线、旅客吞吐量均居中部城市首位;东湖综合保税区封关运行。成功举办全球汽车论坛、世界新兴产业大会、华创会、国际友城高峰论坛等一批国际性会议。

　　——城乡一体化发展势头良好。6个新城区经济实力快速提升，地区生产总值增速高于全市平均水平2.2个百分点;地方公共财政预算收入增长21.5%，高于全市平均水平3.4个百分点。“菜篮子”工程提档升级，新建7.4万亩设施蔬菜基地。农产品加工业产值突破2000亿元。新建等级公路126公里、通湾公路530公里。

　　——人民生活持续改善。全市公共财政用于民生领域的支出达703.4亿元，增长26.5%;民生支出占全市公共财政支出比重为63.7%，比上年提高1.5个百分点。预计城市居民人均可支配收入突破30000元，农民人均纯收入12500元。教育、文化、体育、医疗卫生、社会保障等公共服务水平稳步提高，市容环境不断改善，市民生活质量不断提升。

　　一年来，我们主要抓了六个方面的工作。

　　(一)沉着应对经济下行压力，稳增长调结构取得积极成效

　　出台稳增长系列政策措施。制定实施“工业保增长16条”、“激活内需提振商业15条”、“全民创业20条”等系列措施。落实结构性减税政策，停止18项事业性收费。开展“千名干部进千企”服务活动，帮助企业解决实际困难和问题。经济逐季回升向好。

　　深入推进工业倍增计划。优化工业布局，编制大光谷、大车都、大临空、大临港“四大工业板块”空间发展规划;6个新城区和3个跨三环线中心城区，分别建成20平方公里、10平方公里以上工业倍增发展区，全市新增工业园区面积80平方公里。持续加大工业投入，工业投资突破2200亿元，增长30%以上。上海通用武汉生产基地、力诺武汉生产基地、周大福珠宝文化产业园、东风乘用车新能源工厂、东风格特拉克变速箱、格林美产业园、百杰瑞新材料、武桥海工装备产业园等13个投资50亿元以上重大项目建设扎实推进。近两年签约的122个投资5亿元以上的工业项目开工率达到80%。调整优化工业结构，完成工业技术改造投资350亿元，汽车、光电子信息和重化工产业链不断完善。全球10大汽车制造厂商，4家在汉投资合作，武汉成为我国拥有车系最多的城市。规模以上工业增加值突破3000亿元;企业利润总额480亿元，增长32%。

　　加快实施服务业升级计划。金融、现代物流、会展、文化创意、信息、现代商贸等现代服务业加快发展。区域金融中心建设步伐加快，引进各类金融机构8家，累计178家;“新三板”挂牌企业超过40家，获批国家消费金融试点城市，社会融资规模3336亿元，增长10.7%。出台物流业空间发展规划，“一港、六园、八中心”建设加快推进，引进菜鸟网络、敦豪、普洛斯等6家国内外知名第三方物流企业，社会物流总额突破2.2万亿元。成功举办全国糖酒商品交易会、全国药品交易会、中国国际商用车展览会、中国国际农产品交易会等国家级会展，中国汉口北商品交易会、“中国光谷”国际光电子博览会、中国武汉金融博览会、武汉国际时装周等武汉会展品牌影响不断扩大，会展总数突破600场次，吸引境外参展参会人员200多万人次。工程设计产业实现收入800亿元。国内9大电商在汉设立物流配送中心或研发营运中心，电子商务交易额1720亿元，增长40%。软件及信息服务业主营业务收入900亿元，增长38%。旅游总收入1680亿元，增长20.4%。全国旅游标准化示范城市、国家智慧旅游试点城市建设加快推进，东湖生态旅游风景区晋升国家5A级景区。

　　(二)深入推进改革开放，城市发展活力不断增强

　　加强服务型政府建设。推进政府机构和职能调整，整合组建城市管理委员会、卫生和计划生育委员会、文化新闻出版广电局;食品药监、工商、质监管理体制由市垂直管理，调整为市、区分级管理。警务机制改革深入推进，74%的警力下沉到基层。深化行政审批制度改革，市级行政审批事项由339项减为259项，向区新下放行政审批事项23项;建成市、区联动网上并联审批系统，审批服务事项提前办结率达到93%。全面推进依法行政。制定出台武汉市法治政府建设规划。自觉接受市人大及其常委会的法律监督、工作监督和市政协的民主监督，提请市人大常委会审议通过地方性法规草案6件，制定政府规章13件，人大议案、代表建议和政协建议案、提案1379件全部办结。

　　培育壮大市场主体。推进国企改革重组。深化与央企对接合作，新增签约项目22个，长江通信与武汉邮科院战略重组全面完成。支持武商、中百、武汉肉联等优势企业跨区域扩张。市国资委出资企业资产总额达到4600亿元，净资产1200亿元，分别增长22.5%和22.7%。实施中小企业成长工程，加快发展民营经济。新登记私营企业3.13万户、个体工商户7.41万户。13家民营企业入选中国民营企业500强，增加3家。民间投资占全社会固定资产投资比重达58.8%。

　　深化“两型”社会建设综合配套改革。出台生态文明建设目标体系及考核办法、实施循环经济促进法办法。在全国率先出台湿地保护区生态补偿办法。武汉节水型社会试点通过国家验收。生活垃圾分类减量试点启动实施。完善环境监管机制，实现所有湖泊水质监测全覆盖，实时公开PM2.5等环境空气质量监测数据。“两型”社会创建示范工程深入推进。青山工业区获批国家循环化改造示范试点园区，新能源汽车示范运营规模达到1098台，新投放便民自行车1万辆，新增绿色建筑200万平方米。全面完成省下达的节能减排目标任务。

　　制定实施国际化水平提升计划。加快发展开放型经济。招商引资总额2765亿元，增长61%;实际利用外资52.5亿美元，增长18.1%。签约引进投资50亿元以上工业项目12个、30亿元以上服务业项目10个。对外贸易恢复增长，出口总额116.3亿美元，增长8.2%。对外投资、国际工程承包扩大到50多个国家和地区。国际大通道进一步拓展。扩大对外交往合作，友好城市增加到20个，友好交流城市增加到24个;“双谷双城”战略合作进一步深化;中法合作共建可持续生态新城项目落户武汉;法国马赛大学和武汉理工大学签署合作办学框架协议。营造国际化城市环境，外国领事馆区规划建设积极推进，长江国际学校投入使用。

　　在扩大对外开放的同时，扩大对内开放。深度融入“两圈两带”和长江中游城市群建设，首届长江中游城市群省会城市会商会在汉召开，产业、交通、科技等领域区域合作取得实质性进展。

　　(三)制定实施自主创新能力提升计划，国家创新中心建设全面推进

　　深化科技体制改革。完善科技成果转化机制，出台“黄金十条”实施导则，东湖国家自主创新示范区获批开展研发费用加计扣除、科技成果处置权和收益权改革试点。制定实施支持大学生创新创业“青桐计划”，评选100名创业先锋，新增科技企业孵化器123万平方米，建成大学生创业园区26家，400个大学生创业团队入驻，百万大学生创业激情进一步激发。推进科技金融创新，光谷“资本特区”建设加快。新增创投机构60家，累计210家，基金规模超过200亿元。新增科技支行4家，累计14家;股权质押、知识产权质押等创新性融资超过300亿元，科技保险保额达370亿元。深入实施“黄鹤英才计划”、“光谷人才计划”等人才工程，新引进海内外高层次创新创业人才260名，入选国家“千人计划”累计达170人。

　　完善以企业为主体的技术创新体系。新增各级创新型企业45家、工程技术研究中心50家、企业技术研究中心19家。依托龙头企业，新组建产业技术创新战略联盟10家。新设立企业院士工作站5家。依托高校重点学科、优势学科，新组建5家产业技术研究院。新引进一批国内外知名企业研发机构，世界500强企业在汉设立研发机构累计20家。技术创新能力不断增强，涌现出全球首台光功率热分析仪、国际领先的精密重力测量研究设施等一批国际先进水平的自主创新成果，30项科技成果获国家级奖励。发明专利授权量3150件，国家知识产权审查协作中心在光谷正式运行。

　　加快发展战略性新兴产业。实施高新技术产业五年行动计划和战略性新兴产业超倍增计划。启动设立100亿元的战略性新兴产业发展引导基金。全市在建和筹建的战略性新兴产业项目231个。霍尼韦尔涡轮增压器、湖北宽带研发中心、中联药业中药产业化基地等一批项目开工，雅图中国光影城、华灿光电三期等项目加快建设。地球空间信息产业入选全国首批创新型产业集群试点。高科技园区加快发展，世界500强企业中的15家生物医药企业，8家入驻光谷生物城;17家大型央企入驻武汉未来科技城。战略性新兴产业产值2100亿元，增长25%。

　　(四)加大“三农”工作力度，城乡统筹发展取得新进展

　　新城区“四化”同步发展。以工业为主的经济结构初步形成，工业增加值占生产总值比重达到47%，比上年提高5个百分点。新型城镇化稳步推进。按照“独立成市”理念，编制完成6个新城区现代新城近期规划实施纲要和区域城镇体系规划。新城区主城建设积极推进。阳逻、吴家山、纸坊、知音、盘龙、纱帽等新城产城融合步伐加快。实施村镇建设项目219个。武湖街、五里界街、奓山街成为全省“四化”同步试点示范乡镇。现代都市农业加快发展。新增农业标准化生产基地12万亩、国家级农业标准化示范区3个。10大赏花游核心项目启动建设。乡村休闲游游客突破2000万人次。新建林果产业基地4万亩。推广农业新品种、新技术、新模式、新设施116项，推广农机新机具1.9万套，农业机械化水平达64%。新组建农民专业合作社510家、土地股份合作社30家、家庭农场702个。

　　全面推进农村资源资本化。完成15个街乡镇农村产权确权(变更)登记工作。农村产权交易额22亿元。农村土地、林地、水域经营权抵押贷款11.25亿元。新增农村土地流转面积10.64万亩。

　　改善农村生产生活条件。加强农田水利建设，更新改造181处骨干排灌泵站，除险加固40处病险水库，改造9万亩重点易旱地区水利设施。扎实开展“万名干部进万村洁万家”活动，新建农村垃圾转运站17座，29个村庄成为省级宜居村庄。提档升级609家“农家店”，新建500个“村邮站”。各级财政投入革命老区建设资金38亿元，老区生产生活条件不断改善。为140万武汉籍外出务工农民购买人身意外伤害保险。新建10个农村留守儿童关爱服务站。

　　(五)坚持建管并重，城市功能环境实现新提升

　　强化规划先导作用。认真谋划武汉2049远景发展战略。完善“三镇三城”实施性规划体系，制定出台都市发展区1∶2000基本生态控制线规划、国家中心城市重点功能区体系规划。编制完成公共停车场近期实施规划、社区和养老服务设施空间布点规划、普通中小学布局规划、医疗卫生设施空间布局规划等一批专项规划。新版市域国土规划“一张图”系统正式启用。

　　大力推进城建攻坚计划。创新城市基础设施建设体制，变“以市为主”为“市区联动、共同推进”，充分发挥市、区两级积极性和资源优势。基础设施建设投资1380亿元，城建重大项目竣工6个、开工18个、续建38个。

　　交通枢纽建设取得新进展。天河机场三期、航空总部区全面开工。武汉至咸宁城际铁路建成通车。纱帽港区公用综合码头基本建成。机场第二通道、阳逻集装箱三期、江北铁路一期加快建设。武深高速武汉段、汉江海事搜救中心开工建设。

　　城市路网建设加快。长江大道一期、解放大道下延线示范段、姑嫂树路快速通道主线工程完工，八一路地下通道建成通车。轨道交通6号线一期、7号线一期、8号线一期，四环线南段、三阳路长江隧道、慈天公路跨府河大桥，三环线西段北段、东风大道、国博大道、长丰大道、墨水湖北路改造工程开工建设。轨道交通3号线一期、4号线二期、1号线汉口北延长线，四环线西段、鹦鹉洲长江大桥、江汉六桥、东湖通道、杨泗港快速通道四新段、雄楚大街改造、龙阳大道改造等重大项目加快推进。建成微循环道路25条、慢行交通系统80公里。

　　市政设施不断完善。启动实施中心城区排水设施建设三年攻坚行动计划，新建地下排水管网115公里，清淤排水管网4000公里，完成20个易渍水社区整治,城市排渍能力明显提升。主城区污水全收集全处理五年行动计划加快实施，完成龙王嘴污水处理厂改扩建主体工程、化工区污水处理厂一期和黄陵污水处理厂一期工程，新增污水管网191公里。青山堤防综合改造工程启动实施。新增光纤到户覆盖用户30.5万户。

　　“三旧”改造有序推进。汉正街、二七片、街道口、尤李村等重点片区改造进展顺利。古田老工业区纳入全国新一轮老工业基地搬迁改造示范区。武汉获批全国城镇低效用地再开发试点城市。

　　纵深推进“城管革命”。创新城市管理体制机制。《武汉市城市综合管理条例》正式施行。制定出台《武汉市城市管理相对集中行政处罚权办法》。将市规划、建设、水务、环保、食品药监等10多个部门中涉及城市管理的综合执法权归并到市城管委。推进管理重心下移，将涉及城市综合管理的市容、环保、园林绿化、公共基础设施等管理事项的审批权、执行权及相应财权，全部下放到区。数字化城市管理系统实现全覆盖。推进文明创建工作，开展“城管革命”战线行、社区行、校园行，“全民城管”氛围进一步增强。

　　全面启动国家卫生城市和国家环保模范城市创建工作。路面清洗、楼顶垃圾、渣土污染、占道经营、房屋立面、违法建设、集贸市场、小餐饮小作坊、室外炭火烧烤、机动车冒黑烟、铁路沿线环境等专项治理取得明显成效。一批周期性堵点交通拥堵状况得到缓解。“公交都市”建设开局良好。轨道交通与公交线网实现无缝衔接。新开26条微循环公交线路。新建2个公交首末站、100座新式公交候车亭。大汉阳地区现代有轨电车试验线工程开工建设。修复破损人行道16万平方米。新建公厕100座。安装休闲长椅2000条。建成景观花街59条。实施楼宇景观亮化201栋。编制主城区老旧住宅区物业服务改善实施规划。制定改善空气质量行动计划。中心城区和新城区核心区全面禁鞭。完成三环线内365台燃煤锅炉污染综合治理。

　　加强生态保护建设。第十届园博会筹备工作扎实推进。91公里三环线绿化带基本建成，沙湖公园、汉江公园一期建成开放，洪山广场景观恢复提升工程全面完工，新建绿道260公里，建成“三小”绿地和园林小景67个，完成117条道路绿化改造提升。园博园、戴家湖公园、竹叶海公园开工建设。张公堤城市森林公园加快建设。新增建设绿地808万平方米。人工造林6.74万亩。21座破损山体生态修复启动实施。中心城区40个湖泊实现“一湖一档”动态管理，栽设2000多个蓝线界桩，新建30公里环湖路。龙阳湖、墨水湖、黄家湖截污工程和南湖清淤一期工程完工。新城区湖泊“三线一路”保护规划编制工作全面展开。梁子湖水质良好湖泊生态环境保护试点深入推进。沉湖湿地列入国际重要湿地名录，藏龙岛湿地获批国家湿地公园。建成127个市级生态村。

　　(六)惠民生、促和谐，社会事业全面进步

　　加强就业和社会保障工作。城镇新增就业17.6万人，转移农村劳动力5.2万人;扶持自主创业2.5万人。社会保险净增参保42万人次，综合覆盖率97%。城乡居民大病保险启动实施。城乡居民社会养老保险基础养老金提高20%，企业退休人员基本养老金增长11%，3.8万名被征地农民每人每月增发基础养老金200元。发放救助资金9.3亿元，困难群众救助标准平均提高15%以上。建设筹集保障性住房5.98万套，分配入住2.71万套。新建社区居家养老服务中心(站)116家、农村老年人互助照料中心31家，新增养老床位5570张，养老服务信息平台实现社区全覆盖，发放“一键通”手机20万部。

　　提升公共服务质量和水平。优先发展教育事业。新增1.2万名适龄幼儿入园学位。50所公办幼儿园新改扩建、105所小学标准化建设、149所农村学校食堂建设达标工程全面完成。义务教育区域基本均衡省级督导评估顺利通过。职业教育、高等教育不断发展。国家“教育云”试点“班班通”工程覆盖全市中小学校所有班级。落实国家各类助学资金1.7亿元，资助家庭经济困难学生14.1万人次。

　　制定实施国家医疗卫生服务中心发展规划。新增4家三级医院，30家社区卫生服务中心、20家乡镇卫生院实现提档升级。深化医药卫生体制改革，新型农村合作医疗参合率99.9%，政策范围内住院费用报销比例达75%以上;新增基本药物149种，基本药物平均销售价格下降36%;组建大医院直管社区卫生服务机构区域医疗联合体28个、社区家庭医生团队391个，更多居民就近享受便捷廉价优质的医疗服务。名院名科名医名中心培育工程积极推进。人口计生公共服务体系不断完善。

　　深入推进“文化五城”建设。全民阅读活动广泛开展，新增25个24小时自助图书馆。市直文艺院团全年演出2304场。“武汉之夏”获评全国首批公共文化服务优秀示范项目。一批文艺精品获国家级奖励。新改扩建35个室外社区青少年体育活动中心，新增体育场地15万平方米。成功举办中国金鸡百花电影节、琴台音乐节、亚洲跳伞锦标赛、东湖国际名校赛艇挑战赛、市九运会等大型文体活动。启动青岛路—吉庆街片历史文化风貌街区保护改造工程。盘龙城国家考古遗址公园、光谷国际网球中心开工建设。世界女子职业网球超五巡回赛长期落户武汉。

　　加强和创新社会管理。“幸福社区”创建全面推进。社区律师、法律援助工作不断加强。培育发展社会组织1620家。制定实施城市荣誉制度，评选表彰首批功勋市民、模范市民、文明市民。食品药品安全监管、信访、安全生产工作进一步加强。“平安武汉”建设取得新成效，武汉被授予全国社会治安综合治理最高奖“长安杯”。

　　协调发展其他各项社会事业。“六五”普法依法治理工作通过中期考核验收。全国“双拥”模范城创建中期迎检顺利通过。对口援藏援疆等援建工作扎实推进。国防动员、国家安全、民防、消防、仲裁、保密、参事、气象工作不断加强，文史、档案、民族、宗教、侨务、科协、地方志工作积极推进，妇女、儿童、老龄、残疾人事业健康发展。

　　市政府承诺的十件实事全面完成。

　　在推进经济社会全面发展的同时，我们按照中央和省委、市委的要求，深入开展党的群众路线教育实践活动。坚决反对形式主义、官僚主义、享乐主义和奢靡之风，政府作风建设不断加强。切实整改一批群众反映强烈的突出问题。全市“三公”经费下降16.5%;全市性大会减少37.6%，节会庆典活动减少31%。深化“治庸问责”，深入开展民评民议、电视问政活动，严肃查处庸懒散和行政不作为、乱作为、慢作为行为。

　　各位代表，过去的一年，在严峻复杂的形势下，我市经济社会实现了持续健康较快发展，来之不易，弥足珍贵。这是党中央、国务院亲切关怀的结果，是省委、省政府和市委正确领导的结果，是全市人民同心同德、克难奋进的结果，是人大代表和政协委员监督、支持、帮助的结果，是社会各界和衷共济、通力协作的结果。在此，我代表市人民政府，向全市广大干部群众，驻汉部队、武警官兵，中央在汉单位和各类驻汉机构，各民主党派、工商联、无党派人士、各人民团体，港澳台同胞、海外侨胞和国际友人，向所有关心支持武汉建设发展的海内外人士，表示衷心的感谢并致以崇高的敬意!

　　在肯定成绩的同时，我们也清醒地认识到，全市经济社会发展仍存在不少困难和问题，主要是：发展不够仍然是武汉最大的实际，工业实力还不够雄厚，新兴服务业和战略性新兴产业规模还不大，经济发展面临做大总量、提升质量双重任务;节能减排压力较大，资源环境约束趋紧，传统产业产品层次和技术水平不高，自主创新能力与科教资源优势还不相称，转方式、促转型任务艰巨;民营经济发展相对滞后，国有经济活力需要增强，经济外向度不高，深化改革开放更加紧迫;城乡基础设施还不够完善，城市建设任务繁重、资金筹措难度加大;市民反映强烈的交通拥堵、空气污染等“大城市病”和水环境的综合治理机制亟待创新;部分人群就业难和一些企业招工难并存，社会保障水平还不够高，看病贵和教育、医疗资源均衡化问题需要进一步解决，食品安全、安全生产等工作还存在薄弱环节，这些方面与群众的期待和要求还有差距;少数政府部门和工作人员服务意识不强，执行落实不力、办事效率不高的问题依然突出。城市治理体系有待完善，治理能力有待提高。市民期望城市不光要“跑得快”，还要“跑得更漂亮”。这些问题，我们一定高度重视，以更大的决心、更有效的措施，切实加以解决。

　　二、2014年工作建议

　　党的十八届三中全会对在新的时代条件下全面深化改革作出了总部署、总动员，必将对推动中国特色社会主义事业发展产生重大而深远的影响，为武汉加快科学发展、跨越式发展提供了前所未有的新机遇，开辟了新的广阔空间。

　　当前和今后一个时期的政府工作，要把全面贯彻落实好党的十八届三中全会精神作为一项重大政治任务，以改革统领经济社会发展全局。坚持社会主义市场经济改革方向，以促进社会公平正义、增进人民福祉为出发点和落脚点，以经济体制改革为重点，全面、正确、准确、有序、协调推进各项改革，在转变政府职能、科技体制改革、生态文明制度建设、新型城镇化体制机制创新和建立内陆地区对外开放新模式新机制等方面取得决定性成果，构建系统完备、科学规范、运行有效的制度体系，推进城市治理体系和治理能力现代化。不断提升城市价值，推动武汉成为经济发达、实力雄厚的大武汉，知识引领、创新领先的大武汉，智慧便捷、畅通九州的大武汉，宜居宜业、幸福和谐的大武汉，成为中国中部具有重要影响力的国家中心城市，引领形成区域一体、辐射全球的“大武汉都市圈”，促进大武汉的全面复兴。

　　2014年，是全面贯彻落实党的十八届三中全会精神的第一年，是实施“十二五”规划的攻坚之年。政府工作的总体要求是：认真贯彻党的十八大和十八届三中全会精神，坚持稳中求进的工作总基调，按照竞进提质、升级增效的工作总要求，全面启动新一轮改革，把改革创新贯穿于经济社会发展各个领域各个环节，以改革促创新发展，以改革促转型升级，以改革建生态文明，以改革促民生改善，深入实施“五大计划”，着力保持较快发展速度，切实提高发展质量和效益，改革当先锋、转型走前列、发展争进位，为建设国家中心城市和国际化大都市、复兴大武汉奠定坚实基础。

　　经济社会发展的主要预期目标是：地区生产总值突破1万亿元，增长10%;地方公共财政预算收入增长13%;全社会固定资产投资增长16%以上，社会消费品零售总额增长14%左右，居民消费价格涨幅控制在全国、全省平均水平以内;城乡居民收入增速与经济增长速度基本同步;完成省下达的节能减排任务。

　　今年是新一轮改革的开局之年。我们将按照中央和省委、省政府统一部署，在市委的领导下，制定实施武汉全面深化改革行动计划。从最急迫事项改起，从老百姓最期盼的领域改起，从制约经济社会发展最突出的问题改起，从各方面能够达成共识的环节改起，推出一些立竿见影的改革，让老百姓得到实实在在的好处，让全社会感受到市场环境、创业条件、干部作风在一天天好转，以改革的主动赢得发展的主动。

　　重点做好以下九个方面的工作。

　　(一)深化经济体制改革，着力激发市场活力

　　处理好政府与市场的关系，使市场在资源配置中起决定性作用和更好发挥政府作用，努力打造一流的投资环境和创业环境。

　　推进国企民企共同发展。出台新一轮国资国企改革发展总体方案。积极引入非公有资本和战略投资者，参与市属国有出资企业及其子公司的产权多元化改革，大力发展混合所有制经济。推动国有资产监管从管资产为主向管资本为主转变，加快培育有实力的国有资本经营公司，支持有条件的国有企业改组为国有投资公司，推进城建投融资企业转型。分类完善国有企业法人治理结构和激励约束机制，增加国企管理人员市场化选聘比例。

　　激发民营企业创新发展活力。深入落实支持民营经济发展各项政策。废除对非公有制经济各种形式的不合理规定，消除各种隐性壁垒。制定完善政策措施，推动民间资本进入基础设施、基础产业、金融服务和教育、医疗卫生、养老、健康等领域。建立健全中小民营企业信用体系、融资担保机制，缓解民营企业融资难。鼓励有条件的民营企业建立现代企业制度，支持民营企业上市融资。

　　加快转变政府职能。研究制定《武汉市政府职能转变和机构改革方案》。进一步简政放权。做好国家和省取消、下放行政审批事项的承接工作，再取消和下放一批行政审批事项，继续优化行政审批服务流程。清理规范审批中介环节，切实推进中介机构与审批机关脱钩。编制政府购买社会组织服务目录和细则，推广政府购买服务。稳步推进行政类和经营类事业单位改革。优化政府组织结构，推进市场监管、农业农村管理、交通运输、城乡规划建设等领域机构和职责调整。开展公务员聘任制和事业单位职员制试点。

　　有序推进投资、财税体制改革。推动企业投资项目依法依规自主决策。加强政府投资资金的统一管理。改进预算管理制度。实行全口径预算管理体系，稳步推进预算绩效管理;建立债务风险防范和债务偿还机制，逐步将政府债务纳入财政预算管理;完善一般性转移支付增长机制;健全财政专项资金管理制度。加快构建地方税体系，做好“营改增”试点工作。进一步梳理市、区事权，建立事权和支出责任相适应的制度。

　　(二)坚持两业并举，加快产业转型升级

　　坚持把产业调整作为转变发展方式的主攻方向，调整提升制造业，加快发展现代服务业，在规模中求转型，在转型中求规模，促进经济总量和质量效益同步提升。

　　持续推进工业倍增计划。抓工业规模，抓重点产业，抓产业集群。实施工业重点产业链(企业群)构建工程，延伸壮大产业链。启动实施“千企升级三年行动工程”，完成技术改造投资650亿元，加快传统产业改造升级。加大项目建设力度，推进100个投资5亿元以上的重大项目，确保50亿元以上项目开工5个，10-50亿元项目开工10个、投产10个。确保上海通用武汉生产基地、周大福珠宝文化产业园、格林美产业园、力诺武汉生产基地、北车轨道交通修造基地等一批重大项目竣工投产;确保东风雷诺一期、东风本田三厂、卓尔航空、科聚新材料、长飞科技园、中核环保产业园等一批重大项目开工;加快建设东风格特拉克变速箱、武桥海工装备产业园、百杰瑞新材料、霍尼韦尔涡轮增压器、东风史密斯半挂车等一批重大项目。促进工业倍增发展区拓展空间、提升功能，推进大光谷、大车都、大临空、大临港“四大工业板块”建设。工业投资突破3000亿元，增长30%以上。工业总产值达到13000亿元以上，工业增加值突破4000亿元。

　　持续推进服务业升级计划。争取《武汉金融改革创新总体方案》尽快获批实施。新引进8家金融机构，推动设立3家以上消费金融公司、金融租赁公司，在推动设立民营银行上实现突破，全面建成武汉民间金融街。完善多层次资本市场，提高直接融资比重，推动8家以上企业在境内外上市。加快推进物流“一港、六园、八中心”建设，引进4家以上国内外知名物流企业，加快武汉物流交易所和大宗商品交易所建设，物流总额突破2.5万亿元。加快商贸业提档升级。电子商务交易额突破2500亿元，连锁经营销售额突破2000亿元。推进宜家销品茂、永旺梦乐城、欧尚商贸城等重点商贸项目建设。逐步完善武汉国际博览中心配套功能。引进2个以上国际一流展会。推进工程设计之都建设。创建中国软件名城。积极发展科技服务业。推进国家智慧旅游城市试点，通过全国旅游标准化示范城市验收，打造武汉“新花城”和“两江四岸”旅游精品，支持木兰文化生态旅游区创建国家5A级景区。加快武汉中央商务区、武汉中央文化区、汉正街中央服务区、武昌滨江商务区、杨春湖高铁商务区等服务业聚集区建设。制定实施楼宇经济发展规划和扶持政策，建成5A甲级写字楼100万平方米，税收过亿元楼宇达到25栋。

　　(三)深化科技体制改革，加快创新驱动发展步伐

　　大力实施自主创新能力提升计划，提升原始创新、集成创新、引进消化吸收再创新能力;健全技术创新市场导向机制，进一步打通科技和经济社会发展之间的通道，促进科技成果资本化、产业化，加快把科教资源优势转化为经济发展优势。

　　增强东湖国家自主创新示范区的引擎作用。加大先行先试力度，努力把光谷打造为全国科技体制改革综合试验区。认真落实示范区部际协调小组第三次会议精神。参照落实“中关村新四条政策”。开展职务科技成果及其形成的知识产权管理改革试点。深入推进研发费用加计扣除和职工教育经费税前扣除等财税政策试点、股权激励政策试点、国家战略性新兴产业区域集聚发展试点等工作。加快光谷“资本特区”建设。争取设立光谷科技银行。推进环官桥湖“资本谷”规划建设。加强知识产权运用和保护，加快建设光谷“国家知识产权示范园区”。全面推动光谷生物城、未来科技城、光谷中心区等生态型科技新城建设发展。加快建设武汉软件新城、光谷金融港、激光产业园、地球空间信息产业园、文化和科技融合示范基地等一批专业园区。推动示范区先行先试政策辐射到全市，形成“一区多园”格局。

　　强化企业技术创新主体地位。引导支持企业加大研发投入。支持大型企业自主建设研发机构，发挥创新骨干作用;构建一批面向企业特别是中小企业的技术创新服务平台，激发中小企业创新活力。新增一批国家认定的企业技术中心、工程(技术)研究中心、重点实验室。加快推进应用型技术研发机构市场化、企业化改革。健全产学研协同创新机制。新组建市级以上产业技术创新战略联盟5个、企业院士工作站3个，推动产业技术研究院创新体制机制，尽快产生并转化一批应用科技成果。集中力量抓好战略性、全局性、前瞻性的重大创新项目，组织实施关键技术攻关项目75项。创新人才发展机制。探索外籍高端人才绿卡制度，引进海内外高层次人才230名以上。完善人才政策和服务体系，激发人才创新创业活力。

　　健全技术转移机制。加强成果转化机制和平台建设。全面落实湖北“科技十条”和武汉“黄金十条”，建立技术经纪人制度，构建科技成果转化服务大平台，使发明者、创新者能够合理分享创新收益。全面实施“青桐计划”，培育大学生创业先锋，壮大创新创业队伍。新增大学生创业园区20个、科技企业孵化器120万平方米。定期举办“青桐汇”，打造青年创业者与创投资本对接、与创业导师对话、分享创新创业经验的平台，筹建创业培训学院，让“知识青年”转化为新型商业力量。发展技术市场，技术合同交易额达到260亿元。促进科技与金融结合，扩大“新三板”试点，新增挂牌企业30家;新引进各类创投机构60家以上;新增科技贷款150亿元、科技保险保额200亿元。

　　壮大战略性新兴产业规模。抢抓新一轮科技革命和产业变革孕育兴起的机遇，在战略性新兴产业领域，加强科技研究、加快产业布局，抢占未来发展制高点。策划和推进一批投资50亿元以上新兴产业项目。新增高新技术企业100家以上。巩固提升光电子产业竞争优势，壮大生物、地球空间信息、节能环保等产业规模，培育发展物联网、云计算等新兴产业，推进3D打印、工业机器人、生物质能产业化发展。高新技术产业产值增长20%以上，战略性新兴产业产值增长25%以上。

　　(四)推进城市建设管理创新，提升城市功能和形象

　　坚定不移地推进城建攻坚计划。坚持国际视野、一流标准，按照“重基础、重民生、重生态、重文化、重品质”的要求，高起点规划城市，高水平建设城市，高效能管理城市。城市基础设施投资突破1500亿元。竣工14个重大项目，开工建设11个重大项目。

　　完善全域规划体系。落实武汉2049远景发展战略实施意见。启动修编新一轮城市总体规划、建设国家中心城市规划纲要、土地利用总体规划、综合交通规划，启动“十三五”规划研究编制工作。按照“圈层联动、一体发展”思路，完善空间规划体系，加快构建“3+N”城市内部空间结构，加强地下空间开发利用研究。

　　推进城建投融资体制改革。进一步完善市区两级政府共同推进城市基础设施建设体制。更好利用融资租赁、企业债券、股票市场、中期票据、保险资金、“拨改租”等多种方式，筹集建设资金。探索设立城市建设基金。制定非公有制企业进入特许经营领域具体办法，鼓励社会资本参与城市公用设施投资运营。探索建立盘活城市基础设施存量资产的机制。

　　推进铁水公空重大项目建设。加快天河机场三期建设。支持武汉至黄冈城际铁路建成运营，推进武汉至孝感城际铁路建设。加快武汉新港阳逻集装箱三期建设步伐，开工建设林四房港区煤炭码头。推进武深高速武汉段、硚孝高速建设。

　　完善市域快速交通体系。同步推进9条轨道交通线建设。确保1号线汉口北延长线、4号线二期建成运营，实现武汉三镇全部开通地铁。加快建设3号线一期、6号线一期、7号线一期、8号线一期，开工建设机场线、21号线、27号线。加快过江通道、城市环线和快速路建设。建成鹦鹉洲长江大桥、姑嫂树路快速通道、杨泗港快速通道四新段、通顺大道、兴业路延长线，完成三环线西段北段、国博大道、龙阳大道、墨水湖北路、李纸路改造，基本完成雄楚大街改造。实现二环线主线贯通。加快江汉六桥、汉江三官公路大桥、慈天公路跨府河大桥、岱家山大桥、东湖通道、江北快速路、解放大道下延线、古田四路北延线、东风大道、长丰大道、21号公路改造、园博园配套道路等项目建设。开工建设杨泗港、沌口、青山三座长江大桥，以及长江大道二期、滨江大道延长线、新武金堤路等一批项目，启动三环线南段、光谷大道、武咸公路、青王公路改造工程，推进四环线全线开工。编制全域公交专项规划。加快雄楚大街快速公交系统、大汉阳地区现代有轨电车试验线建设。建成国博中心、盘龙城、凤凰山等一批公交枢纽站、首末站。大力发展慢行交通，建成一批自行车道、人行步道。

　　加强市政基础设施建设。坚持地面设施与地下设施并举，统筹推进排水、排污、供水、电力、通讯等设施建设。启动排水深隧、大型排水通道系统规划研究。加快实施中心城区排水建设三年攻坚行动计划。完成长江大道、红庙立交、台北路等一批排水工程，加快后湖四期、港西一期等泵站建设改造，新建排水管网100公里。深入实施主城区污水全收集全处理五年行动计划。加强二郎庙、落步咀、龙王嘴、黄金口等重点区域污水收集管网建设，新建污水管网100公里。推进汉西、南太子湖、三金潭等污水处理厂改扩建工程，完成汤逊湖污水处理厂扩建主体工程。建成金口自来水厂一期工程，完成余家头自来水厂扩建。推进危险废物处置中心建设。建设4G移动通信网络。

　　统筹推进城市更新改造。加快旧城区、旧厂房、旧村落和棚户区改造步伐，推进长江大道沿线、汉正街、二七片、谌家矶、古田片、龟北片、蛇山北片、白沙洲片等区域成片开发。

　　推进“城管革命纵深行”。升华理念，提高标准，健全制度，深入落实《武汉市城市综合管理条例》，全面提升市容管理、绿化美化、交通管理、生态环境治理水平和市民文明素质。

　　深入推进“双创”工作，实现国家卫生城市创建全面达标，达到省环保模范城市创建要求。深化城市综合管理执法体制和考评机制改革，推进环卫作业市场化改革。完善全天候、全覆盖的清扫保洁网络，巩固提升主次干道、窗口地带保洁水平。强化城市进出口道路、铁路沿线、集贸市场、背街小巷、城乡结合部、村级工业园等薄弱区域环境综合治理，整治规范占道洗车、占道汽修等占道经营行为，实现违法建设“减存量、零增量”。实施房屋全生命周期管理，建立动态的房屋基础数据库。推进主次干道、历史街区临街房屋立面和门面招牌整治。连片整理规范架空管线。深入推进园林绿化管理法制化、标准化、精细化、长效化。建立完善城市交通综合协调机制，强力整治交通秩序。加强城建工程施工管理，尽可能减少对交通的影响;强化电动自行车综合治理，依法禁止超标电动自行车在主城区销售、通行;严厉查处渣土运输车交通违法行为;规范非机动车停放秩序。

　　积极创建全国文明城市。大力实施市民素质教育提升工程，深入开展文明交通行动、周末清洁家园行动，进一步调动广大市民参与城市管理的积极性、主动性。

　　(五)推进新城区“独立成市”，促进城乡共同繁荣

　　健全城乡一体化发展体制机制，积极稳妥推进新型城镇化，推动6个新城区建设主城明确的城镇体系、各具特色的产业体系、相对完备的基础设施体系和公共服务体系，加快形成自成一体、功能完善、产业实力强、中等规模的现代化新城。

　　着力提高城镇化质量。编制实施《武汉市新型城镇化暨全域城乡统筹规划》，推进新城区产业现代化、功能现代化、城市格局现代化。引导工业大项目向产业新城聚集，促进产城融合发展。加快建设污水处理厂、环卫垃圾处理、消防、给排水等城镇配套基础设施和公共服务设施。有序推进农业转移人口市民化。推进武汉经济技术开发区与汉南区一体化融合发展，继续抓好汉阳城乡统筹示范区、武汉化学工业区等新型城镇化改革试点。协调推进城镇化和新农村建设，慎砍树、不填湖、少拆房，尽可能在原有村庄形态上改善居民生活条件。积极开展生态乡镇、生态村“四级联创”，推动东西湖、蔡甸等区创建国家级生态区。加快革命老区建设发展，按市定标准，新增脱贫人口8000人以上。

　　深化农村综合改革。加快构建新型农业经营体系。培育200个农民专业合作社、840个家庭农场，探索建立农业资金互助合作社，制定鼓励工商资本到农村发展现代种养业的政策措施。深化农村产权制度改革，赋予农民更多财产权利。启动31个街乡镇农村产权确权登记，增加农村产权交易品种。推进农业资源资本化，组建土地股份合作社50家，探索水面使用权、农业设施等农村产权抵押融资。

　　提高农业现代化水平。以工业化理念谋划农业，以市场化、产业化、组织化的方式抓农业。加强农业科技创新。推进“菜篮子”工程提档升级，新增农业标准化生产基地7万亩。规划建设一批具有一定规模的现代农业示范园。大力发展农产品精深加工，农产品加工业产值突破2300亿元。加快发展以赏花经济为特色的乡村休闲旅游业，新增一批省级旅游名村。结合“万名干部进万村惠万民”活动，加强农田水利基本建设，扎实开展“清万渠”行动。

　　(六)加快生态文明建设，推进绿色循环低碳发展

　　牢固树立尊重自然、顺应自然、保护自然的生态文明理念，增强绿色发展的紧迫性、自觉性和刚性约束，在发展中保护，在保护中发展，彰显武汉独特的山水资源禀赋，让市民“望得见山，看得见水，记得住乡愁”，让武汉的天更蓝、地更绿、水更清。

　　深化生态文明体制改革。深入推进“两型”社会综合配套改革。严格执行武汉市都市发展区基本生态控制线规划、武汉市基本生态控制线管理规定;按照“促进生产空间集约高效、生活空间宜居适度、生态空间山清水秀”的总体要求，制定出台主体功能区实施计划，全面划定和严守生态保护红线。推进府河、武湖、大东湖、汤逊湖、青菱湖、后官湖6条生态绿楔建设，严格锁定三环线生态隔离带。建立健全资源有偿使用和生态补偿制度。大力发展环保市场，深入推进节能量、碳排放权、排污权交易试点，积极推进环境污染第三方治理。扩大绿色信贷和绿色保险试点。建立完善垃圾分类回收和处理制度。健全生态环境保护责任追究制度。

　　强力推进节能减排。加强重点领域节能减排和监管，加快推进重点用能单位能耗在线监测。实施节能改造、节能技术产业化示范等重点工程。推广应用新能源汽车和高效节能产品。推进企业清洁生产。全面实施居住建筑节能65%的设计标准，新增绿色建筑200万平方米、可再生能源建筑应用400万平方米。积极推进国家“低碳城市”试点。

　　大力发展循环经济。深入推进东西湖、青阳鄂循环经济示范区建设，加快产业园区循环化改造。开展机床、工程机械等再制造试点。加快构建餐厨废弃物处理体系。实施《推进武汉市大宗固体废弃物资源综合利用行动方案》。

　　建设城市绿地生态体系。抓紧推进园博会筹备工作。加快建设园博园、张公堤城市森林公园、张毕湖公园、竹叶海公园，建成戴家湖公园、龙灵山生态公园。继续推进“两江四岸”江滩建设。启动建设龟山“城市阳台”。推进长江大道、汉阳大道等主干路景观大道建设，提升100条城市道路绿化景观。新建39个城市“三小”绿地和园林小景。编制山体保护规划，加快破损山体生态修复和环山道路建设，提升九峰山、马鞍山等城郊森林公园生态功能。新增建设绿地500万平方米，建设绿道220公里，植树60万株。

　　深入实施“碧水蓝天”工程。加快推进水生态修复工程。编制完成新城区湖泊“三线一路”保护规划。启动实施环湖泊公园建设工程，形成“一湖一景”。积极推进湖泊截污，加强南湖等湖泊综合治理。加强沉湖、涨渡湖等湿地自然保护区建设，维护湿地原生态。严格保护长江、汉江、梁子湖等战略水源地安全。严厉打击违法填湖、违法排污行为。实施改善空气质量行动计划，综合施策治理雾霾。完成二环至三环内燃煤锅炉污染整治，基本建成高污染燃料禁燃区。全面实施机动车环保定期检验和环保标志管理，扩大“黄标”车限行范围。严格控制建筑工地、城市道路扬尘污染。积极参与武汉城市圈大气污染联防联控联治专项行动。

　　(七)推进社会事业改革发展，切实保障和改善民生

　　坚持保基本、补短板、兜底线，突出重点、完善制度，尽力而为、量力而行，下大力气解决群众最关心最直接最现实的利益问题，努力提供多样化公共服务，让孩子们快乐成长，青年人乐业创业，老年人颐养天年，市民生活更加幸福、更有尊严。

　　提升就业和社会保障水平。健全促进就业创业体制机制，突出抓好高校毕业生等重点群体就业，深入推进全民创业。培育扶持自主创业者2.5万人，创业带动就业11万人。城镇新增就业15万人，农村劳动力转移就业5.5万人。建立更加公平可持续的社会保障制度。扩大社会保险覆盖面，净增参保30万人次。妥善解决被征地农民参加社会养老保险问题。启动医疗保险费用支付方式改革。研究建立基本养老金正常调整机制，同步提高其它社会保险待遇。建设筹集保障性住房8.3万套，建成4.8万套，分配入住3.5万套。积极应对人口老龄化，建立健全社会养老服务体系。推进“养医结合”，提升社区养老服务的医疗、康复、护理功能，让老人干干净净活出尊严、舒舒服服活出品质。

　　大力发展教育事业。深化考试招生评价制度、办学体制等教育领域综合改革。启动实施“武汉名校长培养工程”，完善名教师培养选拔机制。制定实施新一轮学前教育三年行动计划，创建省级学前教育示范区。推进义务教育优质均衡发展、普通高中优质多样化发展。大力发展现代职业教育，推行订单培养。支持各类高校提升发展水平。深入推进国家“教育云”试点工程。

　　增强城市文化软实力。彰显城市文化底蕴，主动融入世界文化，提升城市文化品位。深化文化体制改革。推动政府由办文化向管文化转变，激发文化创造活力。全面完成市直文艺院团改革改制工作。加快“文化五城”建设，着力构建现代公共文化服务体系。实施文艺精品工程，创作一批汉派经典剧目。设立“武汉市文化艺术市长奖”。加强汉口老租界、武昌昙华林、汉阳归元片等历史文化风貌街区的整体规划保护，延续城市历史文脉。建成开放江汉关博物馆、汉秀剧场，加快推进盘龙城国家考古遗址公园、光谷国际网球中心、世界中学生田径锦标赛场馆、武胜国际文化城建设，开工建设武汉国际赛车场。组织好世界女子职业网球超五巡回赛、琴台音乐节、国际风筝邀请赛等大型文体活动。加强学校体育工作，广泛开展全民健身运动。

　　提高人民健康水平。深化医药卫生体制改革。推进公立医院管办分离，鼓励引导社会资本参与公立医院改制重组。有序推动基本药物制度逐步延伸到非政府办基层医疗卫生机构。鼓励社会办医，优先支持举办非营利性医疗机构。推进大医院直管社区卫生服务机构，壮大区域医疗联合体，实现医通、人通、财通，形成基层首诊、分级诊疗、双向转诊新机制。健全社区医生和居民契约服务关系。加快建设国家医疗卫生服务中心。推进国家疑难重症专科诊疗中心建设，完善重点专学科建设体系。新增4家三级医院，建设50个中心村卫生室。启动实施一方是独生子女的夫妇可生育两个孩子的政策，逐步调整完善生育政策，促进人口与城市协调发展。

　　加强国防动员和后备力量建设，深化“双拥”共建，推动军民融合深度发展。

　　今年，我们将继续办好十件实事。

　　1、改善交通出行。开通15条微循环公交线路和老武昌公交游览专线，增加公交车辆200台。增设1000个公交站点候车便民长凳。打通10条断头路，建成微循环道路30条。完善提升主城区30条主干道交通管理基础设施。继续为新入学的全日制大学生每人赠送一本市民手册和一张100元的“武汉通”公交乘车卡。

　　2、改善市容环境。连片整治265个社区、60条主次干道架空管线容貌。修复街巷老旧、破损人行道15万平方米。新建公共厕所100座。淘汰更新1026辆“黄标”公交车。

　　3、强化养老服务。全市城乡居民社会养老保险基础养老金增加20%。新建60家、提档升级40家社区养老院。为全市80周岁以上老年人发放高龄津贴。为困难老年人居家养老发放补贴。继续完善社区居家养老服务“一键通”信息系统建设，拓展服务功能。

　　4、提升教育医疗水平。推进100所公办小学标准化学校建设。完善从学前教育到高等教育的资助体系。实施市管公立医院就诊“一卡通”，实现群众就诊检查结果互通共享。提档升级30家基层医疗卫生机构。为本市户籍6周岁以下患白血病、脑瘫等重大疾病，且家庭贫困无力继续承担后续住院治疗费用的儿童，一次性提供5万元以内的人道救助。为新生儿实施免费耳聋基因筛查。

　　5、实施文体惠民。补充更新2157个农家书屋出版物。在新城区部分风景区设立免费开放日。推出和完善15条休闲健身步道。为415支农村基层群众文艺团队配置活动器材。新建30个室外社区青少年体育活动中心、25个室内社区健身俱乐部。在三镇选择35家游泳场所，继续为中小学生提供15天免费暑期游泳，并开展免费游泳培训。

　　6、加强扶残济困。城乡低保标准提高8%。贫困残疾人免费乘坐公交车、轻轨、地铁和轮渡。为贫困重度精神、智力残疾人提供居家护理补贴。继续为1000名耳聋、智障、孤独症等儿童提供专业康复训练补贴。

　　7、便利群众生活。治理规范285家农贸市场，对其中200家破旧农贸市场实施升级改造。新建社区蔬菜便民店200家。为流动人口免费发放30万张“武汉通”式居住证。

　　8、强化食品安全保障。在学校周边、集贸市场周边、老旧社区、城乡结合部等重点区域，规范、设立小餐饮便民服务区50个。对100家集贸市场豆制品等重点食品品种实施规范经营。提档升级100家食品小作坊。向全市4万家小餐饮单位免费赠送食品安全知识手册。

　　9、改善老旧社区基础设施和服务。持续推进100个老旧社区“幸福社区”创建工作，重点完善社区道路、排水、二次供水、公共照明等基础设施。推进200个老旧社区绿化提档升级。续建300个社区消防室。

　　10、改善农村生产生活条件。对1163公里1个流量以上的农村排灌港渠进行整治。新建500个“村邮站”。改造116所农村学校食堂和55所农村中小学厕所。建设100个行政村生活垃圾收运示范工程。新建留守儿童关爱服务站10个，探索建立服务站健康运行机制。

　　(八)加快城市国际化进程，以开放促改革促发展促创新

　　抓住国家扩大内陆地区对外开放的机遇，大力实施国际化水平提升计划，加快承接产业转移和“走出去”步伐，不断提升开放功能，推动武汉对外开放上规模、上层次、上水平，努力建设中部地区对外开放门户城市。

　　构建开放型经济发展新格局。把握国家放宽投资准入的机遇，提高先进制造业和现代服务业领域利用外资规模和质量。引进签约投资50亿元以上工业项目、投资30亿元以上服务业项目各10个，新引进世界500强企业12家。实际利用外资增长18%以上。拓展新兴市场，扩大自主知识产权、自主品牌和高新技术产品出口，外贸出口保持合理增长。推动优势工商企业开展境外直接投资，建立生产基地和销售网络;支持优势工程企业建立海外工作基地，承接国际承包工程。

　　提升对外开放功能。拓展国际大通道，增开武汉至美国、澳大利亚等国际直达航线;加强与“汉新欧”国际铁路货运专列沿线外国铁路企业合作，联合组建铁路物流公司，实现货运专列双向常态化运营。筹建国际快件枢纽站。加快口岸大通关建设，提升东湖综合保税区功能，争取设立武汉新港空港综合保税区。积极推进武汉内陆自由贸易试验区申报工作。

　　营造国际化城市环境。全面启动外国领事馆区建设，协调做好美国驻汉领事馆开办签证业务、英国领事馆开馆服务工作，争取更多国家在汉设立领事馆。深入实施外籍人士“家在武汉”工程，规范公共场所双语标识，加快发展国际化的教育、医疗、文化服务体系，推进国际化社区建设。

　　扩大国际交流合作。策划举办中国武汉全球投资峰会等高层次国际会议。协作办好中法建交50周年系列纪念活动，推进中法合建可持续生态新城项目，设立巴黎中国文化中心“武汉之窗”。新增一批国际友好城市和国际友好交流城市。组织开展“千名市民看友城”活动。深化“双谷双城”战略合作。

　　加强区域合作。深度融入“两圈两带”和长江中游城市群合作，推进产业大对接、交通大联网、市场大统一、创新大驱动、生态大保护、公共资源大共享、城镇化改革大试验，培育“武汉大都会”。

　　(九)建设法治政府和服务型政府，提升城市治理能力

　　经济社会加速转型，要求城市治理方式随之转变。必须坚持以人为本、民生优先的现代城市治理核心理念，创新城市治理模式，加快推进城市治理体系和能力现代化。

　　建设法治政府，推进依法治理。法治是现代城市治理的基本方式。大力推进依法治市，营造政府依法行政、市场主体依法运营、市民依法行为、整个社会依法维权的良好氛围。实施武汉市法治政府建设规划，进一步推进政府工作法制化。深入开展“六五”普法工作。积极开展重点领域行政立法工作。深化行政执法体制改革，推进综合执法和执法力量下沉，做到严格规范公正文明执法。自觉接受市人大及其常委会的法律监督、工作监督和市政协的民主监督，认真执行市人大及其常委会的各项决议和决定，主动听取市政协的意见与建议，切实办理好人大议案、建议和政协建议案、提案。

　　创新社会治理体制，多方参与、系统治理。发挥政府主导作用，鼓励和支持社会各方面参与，实现政府治理和社会自我调节、居民自治良性互动。加快实施政社分开，推进社会组织明确权责、依法自治、发挥作用。启动社会组织孵化计划，新培育发展2000家社会组织。深入推进“幸福社区”创建，切实减轻社区负担,强化社区自治功能。加强社区矫正和社区戒毒康复工作。创新有效预防和化解社会矛盾体制，使群众问题能反映、矛盾能化解、权益有保障。健全公共安全体系。加快建立食品药品质量安全可追溯体系。坚持安全生产警钟长鸣，建立隐患排查治理体系和安全预防控制体系，大力开展建筑工地、道路交通、地下管网、危化品、消防、特种设备等重点领域安全整治，加快推进武汉化学工业区危化品仓库建设，有效防范重特大事故发生。加强农民工安全生产培训。深化警务机制改革，完善立体化社会治安防控体系。充分发挥市民参与城市治理的积极性、主动性、创造性，促进市民在城乡社区治理、基层公共事务和公益事业中依法自我管理、自我服务、自我教育、自我监督。着力提升市民的“武汉认同感”，让市民关注的目光逐渐从自家以及身边的事情，扩展到城市的共同事务，乃至这座城市所立足的国家背景、时代背景中，增强城市共同治理的活力。

　　建设智慧城市，推进数字治理。智慧城市是衡量城市治理现代化水平的主要标志。加快实施智慧城市建设总体规划，建立完善城市治理信息系统，构建智慧应用、智慧产业、智慧运行三大核心体系，促进城市信息的高效传递和智能响应，为治理城市提供及时、准确、科学的服务，降低治理成本，提高治理效率。统筹推进感知基础设施、网络基础设施、云计算基础设施、地理空间基础设施建设。实施智慧交通、智慧城管、智慧市政、智慧医疗、智慧教育、智慧社区等15个专项智慧规划。启动地下管线信息化平台建设。建立市“政务云”服务总平台，提高网上审批服务效率，让市民少跑路、少受累。

　　强化权力运行制约和监督体系，做到干部清正、政府清廉。清理规范市级行政权力和政务服务事项，依法制定公布“权力清单”和“责任清单”。完善政务公开制度，推进决策公开、管理公开、服务公开、结果公开，让监督的阳光照亮权力运行的每一道环节、每一个角落。

　　加强廉洁政府建设。坚持力度统一论，加大威慑力度、严查力度、监督力度，大力推进反腐倡廉。推进源头治理、制度反腐，深化土地出让、工程建设、政府采购、产权交易等重点领域改革。健全惩治和预防腐败体系，以反腐倡廉的实际成效取信于民。

　　健全改进作风常态化制度。把真抓实干作为政府工作的座右铭，突出“干”字当头，推行“马上就办”，以实干促改革，用实绩赢得群众信任。深化“治庸问责”、优化环境工作，促进政风行风进一步好转。巩固党的群众路线教育实践活动成效，坚决反对“四风”，加大整改落实力度，切实将教育实践活动成效转化为惠民利民成果。严格执行《党政机关厉行节约反对浪费条例》，全面落实《党政机关国内公务接待管理规定》。政府性楼堂馆所一律不得新建，财政供养人员只减不增，公费接待、公费出国、公费购车只减不增。压缩一般性支出，把更多财力用于保障和改善民生，在政府自觉过“紧日子”中，让群众过上“好日子”。

　　各位代表!

　　今天的武汉，站在了新的历史起点，面临着跨越发展的重大机遇，迎来了加速复兴的历史窗口。

　　党的十八届三中全会，开启了新一轮改革的大幕，将给我国带来广泛而深刻的变革，推动经济社会发展全面提升，为敢为人先的武汉争创新优势、实现新跨越，提供了难得的历史机遇。习近平总书记视察湖北时，明确肯定“复兴大武汉”，为武汉实现全面复兴注入了强大动力。

　　国家深入实施区域协调发展、扩大内需、创新驱动发展和新型城镇化战略，依托长江打造新经济支撑带，武汉作为中部地区和长江中游的中心城市、全国重要的交通枢纽和科教基地，多重战略机遇叠加，区位、交通、科教、市场等优势将进一步增强和充分释放，加速转化为发展优势、竞争优势。

　　武汉综合经济实力和城市功能不断增强，步入了加速发展的上升期。全市人民建设国家中心城市愿望强烈，干事创业心齐劲足、热情高涨，形成了加快发展的强大气场。

　　复兴大武汉，天时、地利、人和俱备，机不可失、时不我待。我们要全面贯彻落实党的十八大、十八届三中全会和习近平总书记视察湖北武汉重要讲话精神，按照省委、省政府“建成支点、走在前列”的要求和市委的决策部署，勇于担当、奋发有为，抢抓机遇、乘势而上，为早日建成国家中心城市和国际化大都市，实现大武汉的全面复兴而努力奋斗!
